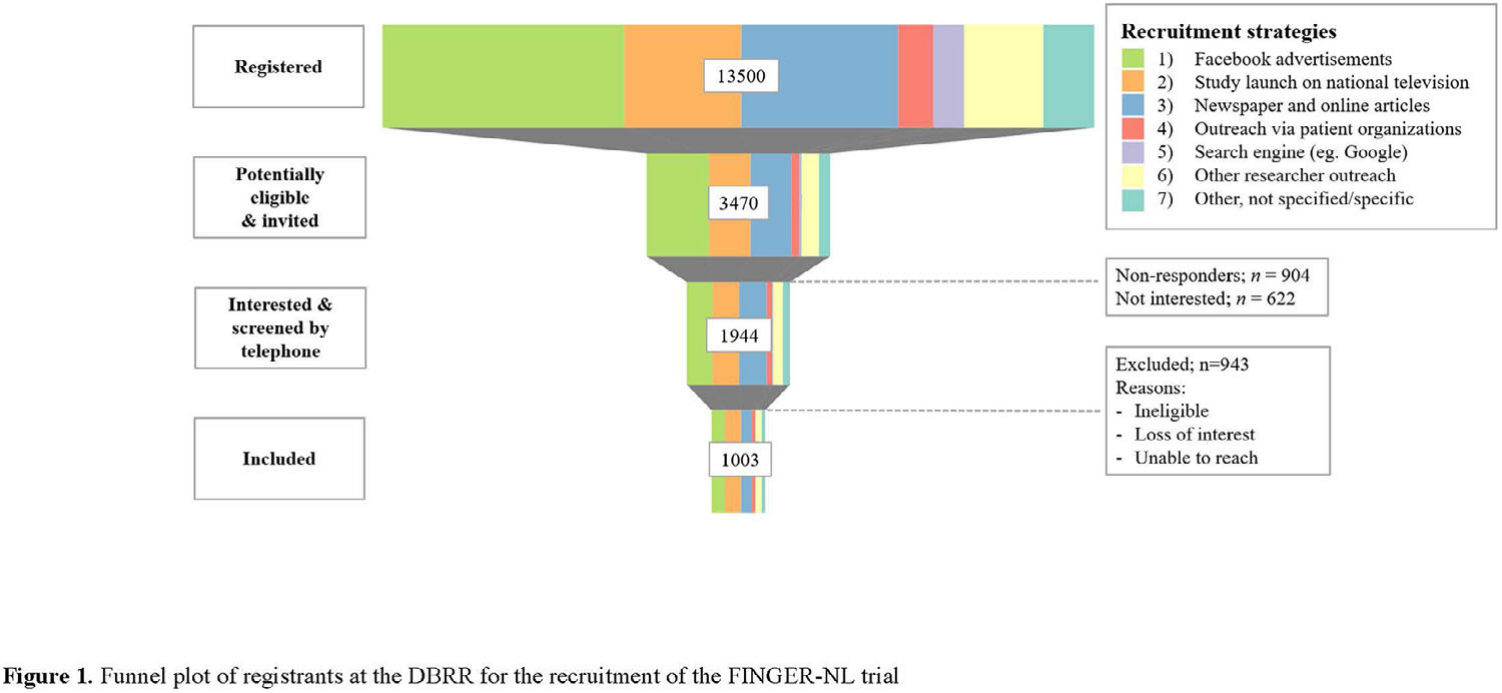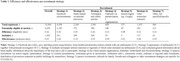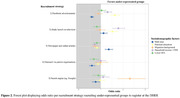# Participant recruitment strategies via the Dutch Brain Research Registry: a comparison of effectiveness and efficiency of different strategies for the multi‐domain lifestyle intervention trial FINGER‐NL

**DOI:** 10.1002/alz.091812

**Published:** 2025-01-09

**Authors:** Lisa Waterink, Sietske A.M Sikkes, Lion M. Soons, Sonja Beers, Ondine van de Rest, Nynke Smidt, Joukje M. Oosterman, Erik J.A. Scherder, Kay Deckers, Lisette C.P.G.M. de Groot, Esther Aarts, Sebastian Köhler, Wiesje M. van der Flier, Marissa D. Zwan

**Affiliations:** ^1^ Amsterdam Neuroscience, Neurodegeneration, Amsterdam Netherlands; ^2^ Alzheimer Center Amsterdam, Neurology, Vrije Universiteit Amsterdam, Amsterdam UMC location VUmc, Amsterdam Netherlands; ^3^ Faculty of Behavioural and Movement Sciences, Clinical Developmental Psychology & Clinical Neuropsychology, Vrije Universiteit Amsterdam, Amsterdam Netherlands; ^4^ Alzheimer Center Amsterdam, Neurology, Vrije Universiteit Amsterdam, Amsterdam UMC, Amsterdam Netherlands; ^5^ Alzheimer Center Limburg, School for Mental Health and Neuroscience, Maastricht University, Maastricht Netherlands; ^6^ Wageningen University & Research, Wageningen Netherlands; ^7^ Department of Epidemiology, University of Groningen, University Medical Center Groningen, Groningen Netherlands; ^8^ Donders Institute for Brain, Cognition and Behaviour, Radboud University, Nijmegen Netherlands; ^9^ Faculty of Behavioural and Movement Sciences, Vrije Universiteit Amsterdam, Amsterdam Netherlands; ^10^ Department of Psychiatry and Neuropsychology, Maastricht University Medical Center+, Maastricht Netherlands; ^11^ Alzheimer Center Limburg, School for Mental Health and Neuroscience (MHeNs), Maastricht University, Maastricht Netherlands; ^12^ Wageningen University, Division of Human Nutrition, Chair group Nutritional Biology, Wageningen Netherlands; ^13^ Department of Epidemiology and Biostatistics, Amsterdam UMC, Amsterdam Netherlands

## Abstract

**Background:**

Recruitment of participants for (lifestyle) intervention studies is challenging, especially when targeting cognitively healthy individuals that need to fulfil specific inclusion criteria. We evaluated the effectiveness and efficiency of a participant recruitment campaign from The Dutch Brain Research Registry (DBRR) for the FINGER‐NL study, a multi‐domain lifestyle intervention trial for older adults. Additionally, we explored which recruitment strategy successfully reached individuals from in research under‐represented groups.

**Method:**

The campaign entailed five active and two passive recruitment strategies: 1) a Facebook‐campaign, 2) appearance on national television, 3) newspaper articles, 4) researcher outreach, 5) outreach via patient organizations, 6) search engines (passive) and 7) other, not specific (passive). Most recruitment strategies, involved a nationally well‐known brain‐health ambassador (E.S.). For each strategy, we describe the number of a) individuals registered, b) potentially eligible, and c) participants included in FINGER‐NL. Subsequently, the efficiency, defined by the eligibility‐ratio (eligible/registered) and effectiveness, defined by the inclusion‐ratio (included/registered) are calculated. Associations between recruitment strategies and sociodemographic factors of under‐represented groups were tested with binomial logistic regressions.

**Result:**

The campaign resulted in 13,500 new DBRR registrants (Figure 1), of which n = 3,470 (eligibility‐ratio 0.26) were eligible and n = 1,003 (inclusion‐ratio 0.07) were included in FINGER‐NL. The Facebook‐campaign and television appearance reached the highest numbers of registrants (n = 4,598 and n = 2,203) and highest number of inclusions (n = 288 and n = 261). The eligibility‐ratio across strategies varied between 0.08 and 0.35 (Table 1), with appearance on national television (0.35), newspaper articles (0.26) and Facebook campaign (0.26) being most efficient. Inclusion‐ratio varied between 0.01 and 0.13. Most effective strategies were national television appearance (0.13), referrals via patient organizations (0.09) and direct recruitment via the researchers (0.08). The Facebook campaign and appearance on national television performed relatively better in recruiting individuals with a practical education and lower household income (Figure 2).

**Conclusion:**

Combining different recruitment strategies benefited the number of included individuals with prevention potential. Mass‐media exposure strategies are efficient in reaching large numbers, and involving an ambassador appears beneficial. Effective strategies contained more information about inclusion criteria and study aim. Recruitment would benefit from targeted strategies for under‐represented groups to improve diversity among research participants.